# *Anopheles aquasalis* transcriptome reveals autophagic responses to *Plasmodium vivax* midgut invasion

**DOI:** 10.1186/s13071-019-3506-8

**Published:** 2019-05-24

**Authors:** Rosa Amélia Gonçalves Santana, Maurício Costa Oliveira, Iria Cabral, Rubens Celso Andrade Silva Junior, Débora Raysa Teixeira de Sousa, Lucas Ferreira, Marcus Vinícius Guimarães Lacerda, Wuelton Marcelo Monteiro, Patrícia Abrantes, Maria das Graças Vale Barbosa Guerra, Henrique Silveira

**Affiliations:** 1 Programa de Pós-Graduação em Medicina Tropical, Universidade do Estado do Amazonas/Fundação de Medicina Tropical Dr. Heitor Vieira Dourado, Manaus, Brazil; 2 Instituto Leônidas & Maria Deane, Fundação Oswaldo Cruz, Manaus, Brazil; 3 Instituto de Higiene e Medicina Tropical, Global Health and Tropical Medicine, Universidade Nova de Lisboa, Lisboa, Portugal

**Keywords:** Malaria transmission, *Anopheles* mosquitoes, Host parasite interactions, *Plasmodium vivax*, Malaria control, Autophagy

## Abstract

**Background:**

Elimination of malaria depends on mastering transmission and understanding the biological basis of *Plasmodium* infection in the vector. The first mosquito organ to interact with the parasite is the midgut and its transcriptomic characterization during infection can reveal effective antiplasmodial responses able to limit the survival of the parasite. The vector response to *Plasmodium vivax* is not fully characterized, and its specificities when compared with other malaria parasites can be of fundamental interest for specific control measures.

**Methods:**

Experimental infections were performed using a membrane-feeding device. Three groups were used: *P. vivax-*blood-fed, blood-fed on inactivated gametocytes, and unfed mosquitoes. Twenty-four hours after feeding, the mosquitoes were dissected and the midgut collected for transcriptomic analysis using RNAseq. Nine cDNA libraries were generated and sequenced on an Illumina HiSeq2500. Readings were checked for quality control and analysed using the Trinity platform for *de novo* transcriptome assembly. Transcript quantification was performed and the transcriptome was functionally annotated. Differential expression gene analysis was carried out. The role of the identified mechanisms was further explored using functional approaches.

**Results:**

Forty-nine genes were identified as being differentially expressed with *P. vivax* infection: 34 were upregulated and 15 were downregulated. Half of the *P. vivax*-related differentially expressed genes could be related to autophagy; therefore, the effect of the known inhibitor (wortmannin) and activator (spermidine) was tested on the infection outcome. Autophagic activation significantly reduced the intensity and prevalence of infection. This was associated with transcription alterations of the autophagy regulating genes *Beclin*, *DRAM* and *Apg8*.

**Conclusions:**

Our data indicate that *P. vivax* invasion of *An. aquasalis* midgut epithelium triggers an autophagic response and its activation reduces infection. This suggests a novel mechanism that mosquitoes can use to fight *Plasmodium* infection.

**Electronic supplementary material:**

The online version of this article (10.1186/s13071-019-3506-8) contains supplementary material, which is available to authorized users.

## Background

Malaria is still an important public health problem in several tropical countries. In 2016, 216 million cases of malaria were diagnosed; Brazil had 18% of all malaria cases that were confirmed by the World Health Organization (WHO) for the region of the Americas [1], and 99.5% of the Brazilian cases were in the Legal Amazon [2] where *Plasmodium vivax* is the predominant species accounting for 85% of reported cases [3].

*Anopheles aquasalis* is an important vector in coastal areas of South America [4, 5]. Since its colonization it has been used for *Plasmodium* experimental infections for research, revealing a robust model to study the interaction of American vectors with *Plasmodium* species [6].

Inside the mosquito midgut the *Plasmodium* gametocyte-to-ookinete-to-oocyst transition is completed within the first 24 hours. The ingested parasite populations suffer substantial losses during this process which corresponds to the most critical population bottleneck of the entire parasite life-cycle; more often than not, transmission is terminated at this stage [7–9]. Invasion of the malaria vector *Anopheles gambiae* midgut by *Plasmodium* parasites triggers transcriptional changes of genes that mediate the antiparasitic defence [10] and, thus, the ability of these mosquitoes to transmit malaria [11]. Moreover, several mechanisms are triggered by the mosquito in order to combat the intracellular pathogen. Apoptosis is one possibility that has been described during ookinete invasion of the midgut [12]. Another related mechanism is autophagy, an important and well-studied cytosolic response. During macroautophagy, a double membrane vesicle called autophagosome forms around cytosolic components, which subsequently fuse with lysosomes and degrade the vessel’s content [13]. Under certain conditions in *Drosophila*, midgut and salivary gland tissues show both high caspase activity and the formation of autophagosomes [14, 15], suggesting that apoptosis and autophagy may be highly integrated in arthropods.

The upregulation of autophagy can enhance resistance to pathogens, a phenomenon that has also been associated with resistance of the fruit fly *Drosophila melanogaster* to bacterial challenge [16] and of mammalian cells to bacteria, viruses and parasites such as *Toxoplasma gondii* [17–21]. Intriguingly, a *P. falciparum* infection in *Anopheles stephensi* induced a translation of autophagy (ATG) protein mRNAs, including those for key regulators ATG6 and ATG8, in the midgut epithelium at 24 hours after infection [22], which suggests that autophagy is induced early during sporogonic development in the mosquito host. While autophagy induction can control resistance, perhaps contributing to the large-scale death of parasites in the midgut, it is possible that highly conserved regulation of stem cell renewal and differentiation by autophagy could also impact the midgut’s response to parasite infection [23].

Transcriptomic analyses of African and Asian mosquitoes in response to pathogens have generated a wealth of data that can facilitate the development of new anti-malaria tools [24, 25]. More recently, *An. aquasalis* specimens have been analysed for functional annotation creating opportunities for further molecular characterization of genes. The *An. aquasalis* transcriptomes of larvae and adults fed on sugar and on blood revealed valuable information about protein-coding transcripts involved in biological processes relevant to mosquito physiology and development of this new world model [26].

Nevertheless, a deeper understanding of the processes taking part on this critical phase of malaria transmission remains unexplored. Here, we report the transcriptional profile of *An. aquasalis* midgut, in the early stage of *P. vivax* development and invasion of the midgut epithelium. *Via* this profile, we were able to gain insights on a molecular level of how to functionally characterize this critical phase of malaria transmission. Our results revealed the importance of alternative mechanisms, such as autophagy, for the control of *Plasmodium* infection in the mosquito.

## Methods

### Mosquito collections and rearing

*An. aquasalis* were obtained from a well-established colony at the Entomology Department’s insectary at the Fundação de Medicina Tropical Dr Heitor Vieira Dourado, Manaus, Amazonas, Brazil (FMT-HVD). All mosquitoes were reared at 26 °C, 70–80% relative humidity under a 12/12 light/dark photoperiod. Larvae were fed with commercial fish food (Tetramin Gold^®^; Tetra GmbH, Melle, Germany) and adults were fed *ad libitum* on 10% sugar solution. Three- to five-day-old adult females were used in all experiments.

### Blood collection

Adult volunteers (ages ≥ 18 years), residents from the region of Manaus (State of Amazonas, Brazil) with *P. vivax* malaria infection diagnosed by blood smears, were recruited at the Fundação de Medicina Tropical Dr Heitor Vieira Dourado (FMT HVD). All volunteers were instructed on the study objectives. A sample of about 10 ml of venous blood was drawn from each patient and placed in heparinized tubes. After blood collection all patients were treated according to Brazilian Health Ministry guidelines [27].

### *Plasmodium vivax* infection of mosquitoes *via* membrane feeding assay

Adult mosquitoes were sugar starved overnight prior to infection and separated into two experimental groups. One group was offered whole blood from *P. vivax* patients for a period of 45–90 min *via* membrane feeding assay (MFA). Blood was held at 37 °C through a hose system connected to a thermal bath [6]. The second group was treated in similar way, but with inactivated gametocytes as described by Mendes et al. [28]: *P. vivax* infective blood was briefly heated for 15 min at 43 °C, chilled to 37 °C and then offered to the mosquitoes. Only fully engorged mosquitoes were transferred to rearing containers and maintained in the insectary at 26 °C with 70–80% relative humidity and fed *ad libitum* on 10% sugar solution.

### Tissue collection and RNA isolation

Mosquito midguts were collected from pools of 30 mosquitoes, 18–24 h after the blood meal, from each of three groups: (i) *P. vivax-*blood-fed group (*Pv*); (ii) non-infective group (*Bl*; blood-fed using *P. vivax*-blood in which the gametocytes were inactivated); and (iii) unfed group (*Unf*; unfed mosquitoes). Tissues were dissected from mosquitoes submerged in ice-cold phosphate buffered saline (PBS) and transferred to RNAlater (Thermo Fisher Scientific, Massachusetts, USA). Samples were stored at − 20 °C until RNA extraction. Total RNA was extracted using TRIzol Reagent (Thermo Fisher Scientific, Massachusetts, USA) following the manufacturer’s protocol. To eliminate possible contaminant genomic DNA, the RNA samples were treated with RNase-Free DNase I according to manufacturer’s protocol (Qiagen, Hilden, Germany). At 8–9 days post-infection, mosquito midguts were collected to determine the infection rate (number of infected mosquitoes over total number of mosquitoes observed) and infection intensity (mean number of oocysts per infected mosquito). Three independent biological replicates of each experiment were performed.

### RNA-seq library preparation and sequencing

The RNA integrity was confirmed using a 2100 Bioanalyzer (Agilent, California, USA). The RNA-seq library preparation and sequencing were performed using total RNA and an Illumina HiSeq^**®**^ 2500 (Illumina, California, USA) at LaCTAD [Life Sciences Core Facility from State University of Campinas (UNICAMP); https://www.lactad.unicamp.br]. Illumina reads from the *An. aquasalis* mosquitoes were checked for quality control using FastQC v.0.11.5 (https://www.bioinformatics.babraham.ac.uk) and analysed using the Trinity platform for *de novo* transcriptome assembly v.2.4.0 [29]. Trimmomatic was used to trim low-quality reads and high quality paired-end reads were assembled using Trinity Assembler v2.4.0 and aligned using Bowtie2 v.2.3.2 [30]. Transcript quantification was performed using RSEM module v.1.2.25 [31]. Transdecoder v.3.0, Trinotate v.3.0.2, BLAST+ (accessed on 2017/04/26) and HMMER v.3.0 searches were used for functional annotation of the transcriptome produced and to populate a Sqlite database.

Differential expression (DE) analysis was performed using GLM test in the *edgeR* v.3.16.5 package [32] in R. Pairwise comparisons were made between the different group samples. In any given group, a transcript was considered differentially expressed if its adjusted *P*-value to control the false discovery rate (Benjamini–Hochberg adjustment) was less than 0.05 and if log fold change was higher than 1. Differentially expressed genes were further analysed for functional classification using gene ontology analysis on PANTHER (http://www.pantherdb.org) [33]. The dataset has been deposited at the Gene Expression Omnibus under the accession number GSE124997.

### Validation of differentially expressed genes

In order to validate transcriptome analysis, a total of 8 differentially expressed genes between mosquitoes fed on blood with infective *P. vivax* (*Pv*) and fed on blood in which gametocytes were inactivated (*Bl*) were chosen for real-time quantitative PCR analysis which was performed as described in [34]. For this, total RNA was used, and first strand cDNA was synthesized using oligo dT and MMLV Reverse Transcriptase (Promega, Wisconsin, EUA) as described in [35]. The cDNA was used as a template for RT-qPCRs using the primer pairs reported in Additional file 1: Table S1. The primers were derived from the sequences identified in the transcriptome. For all groups, mosquito midguts were collected 18–24 h post-infection in order to determine the levels of expression of the genes in the midgut. Experiments were conducted with three biological replicates, each in triplicate.

### Reverse transcription quantitative real-time PCR (RT-qPCR)

Real-time quantitative PCR was performed on a Fast 7500 instrument (Applied Biosystems, California, USA) with SYBR Green Power Master Mix (Applied Biosystems) using 2 μl of cDNA template in a final volume of 20 μl reaction mixture. Fold-changes of gene expression were analysed using the 2^−ΔΔCT^ method. The ribosomal protein S7 was used as the endogenous control.

### Spermidine and wortmannin treatments

The transcriptome associated to *P. vivax* infection revealed a variety of transcripts that play a key role in autophagy. In order to evaluate the effect of the autophagy process in the outcome of infection, we inoculated mosquitoes with wortmannin (an inhibitor of phosphatidylinositol 3-kinase DPI3K) and spermidine (an autophagy activator) [36, 37]. Three- to four-day-old female mosquitoes were cold-anesthetized and inoculated intrathoraxically with 69 nl of a 5 μM and 0.05 μM solution of wortmannin (Merck, Darmstadt, Germany) or with the same volume of H_2_O Ultra Pure and with 69 nl of a 100 μM solution of spermidine (Sigma) or DMSO (0.05%) using a Nanoject micro-injector (Drummond Scientific, Pennsylvania, USA). Twenty-four hours after injection with the solutions, the mosquitoes were fed with a *P*. *vivax-*infected blood meal as described above. Three independent biological replicates were performed for each experiment. Mosquitoes were dissected 18–24 h after feeding; batches of 20–30 midguts were dissected in cold DEPC-treated phosphate-buffered saline (PBS) and processed for RNA preparation and cDNA synthesis using the same protocols mentioned above. Mosquito midguts were also collected on the 8th day post-infection to determine the prevalence and intensity of infection.

### Gene expression of autophagy related genes

The expression of genes that regulate autophagy (*Beclin*, *DRAM* and *Apg8*) was investigated 18–24 h after *P. vivax* infection and 24 h after inhibition and activation of autophagy (treatment with wortmannin or spermidine) as described above.

*Anopheles aquasalis* mosquitoes were dissected 18–24 h after infection; 20–30 midguts were collected, and RNA and cDNA were prepared as described above. Three independent experiments were performed. Gene expression analysis was carried out by quantitative real-time PCR following the same conditions described above.

### Statistical analysis

For data not normally distributed (oocyst density), two-sample comparisons were done using the non-parametric Mann–Whitney test. The differences in the infection rate between the control group and the tested groups were compared using Fisher’s exact one-tailed test (F). Comparisons of mRNA expression levels obtained by RT-qPCR between the control and the tested groups were done using the Mann–Whitney one-tailed test. Statistical analyses were performed using the software GraphPad Prism v.6.00.

## Results and discussion

### General characterization of midgut transcriptome

A total of 9 cDNA libraries from *An. aquasalis* midguts were constructed and sequenced, namely three libraries for each of the following groups: (i) *P. vivax-*blood-fed mosquitoes (*Pv*: groups *Pv1* to *Pv3*); (ii) mosquitoes fed on *P. vivax*-blood from which gametocytes were inactivated (non-infected: groups *Bl1* to *Bl3*); and (iii) unfed mosquitoes (unfed groups: *Unf1* to *Unf3*). The obtained mean number of high quality paired-end short reads were: 56,217,833 (16,351,414–109,481,490), 45,546,489 (44,961,578–46,664,218) and 46,523,955 (43,800,760–50,915,130) for each group, respectively (Table 1).

**Table 1:** Overview of *Anopheles aquasalis* sequencing results

	No. of reads	% bases ≥ Q30
*P. vivax* blood meal (*Pv*)		
*Pv1*	44,961,578	93.16
*Pv2*	45,013,670	90.99
*Pv3*	46,664,218	93.28
Non-infective blood meal (*Bl*)		
*Bl1*	42,820,594	92.15
*Bl2*	16,351,414	93.02
*Bl3*	109,481,490	93.37
Unfed (*Unf*)		
*Unf1*	43,800,760	91.70
*Unf2*	44,855,976	93.36
*Unf3*	50,915,130	90.90

**Fig. 1:**
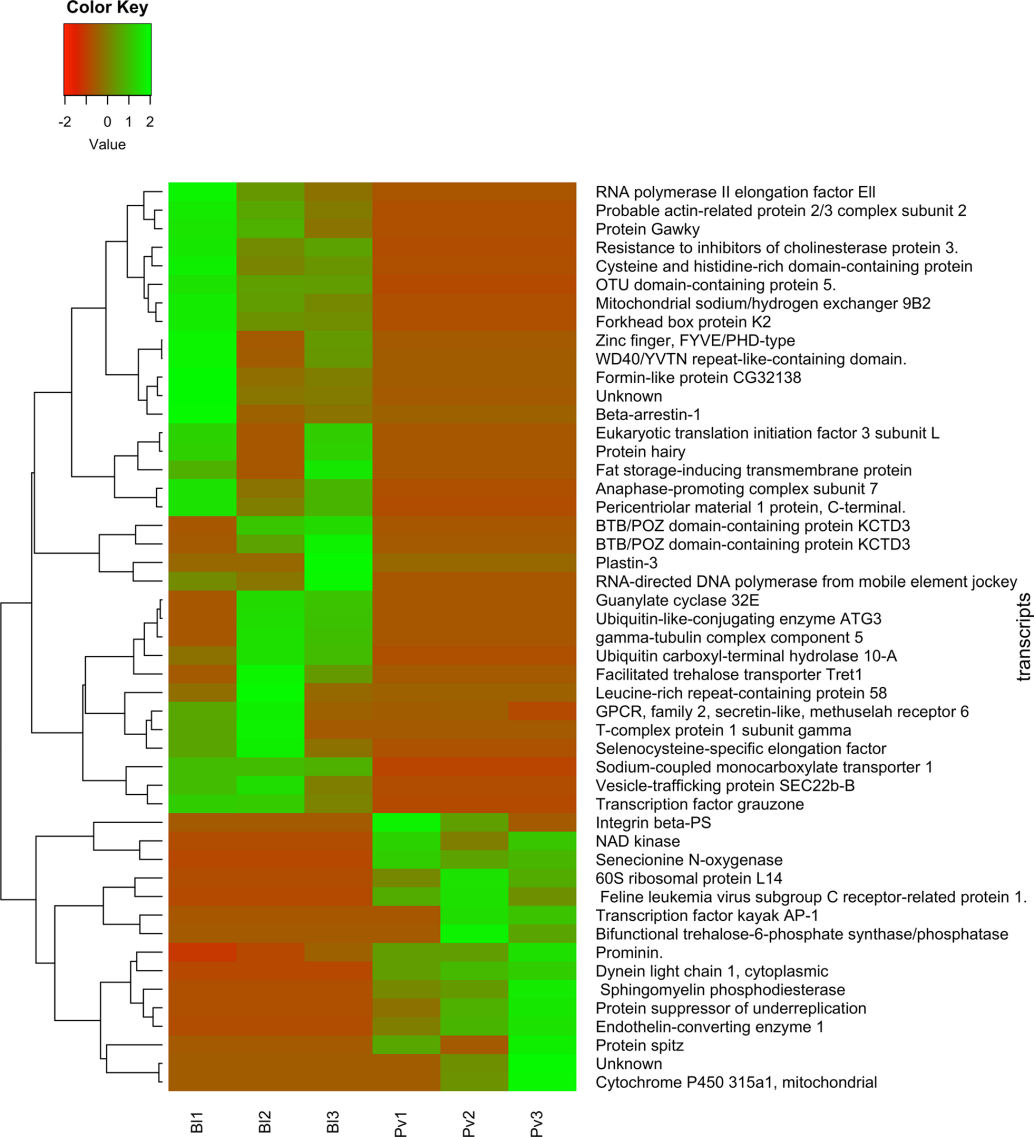
Heatmap showing differential expression of genes between *Plasmodium vivax-*infected (*Pv*) *versus* non-infective blood-fed (*Bl*) groups. Heatmaps were performed with normalized expression values using the *gplots* v.3.0.1 package in R

**Fig. 2:**
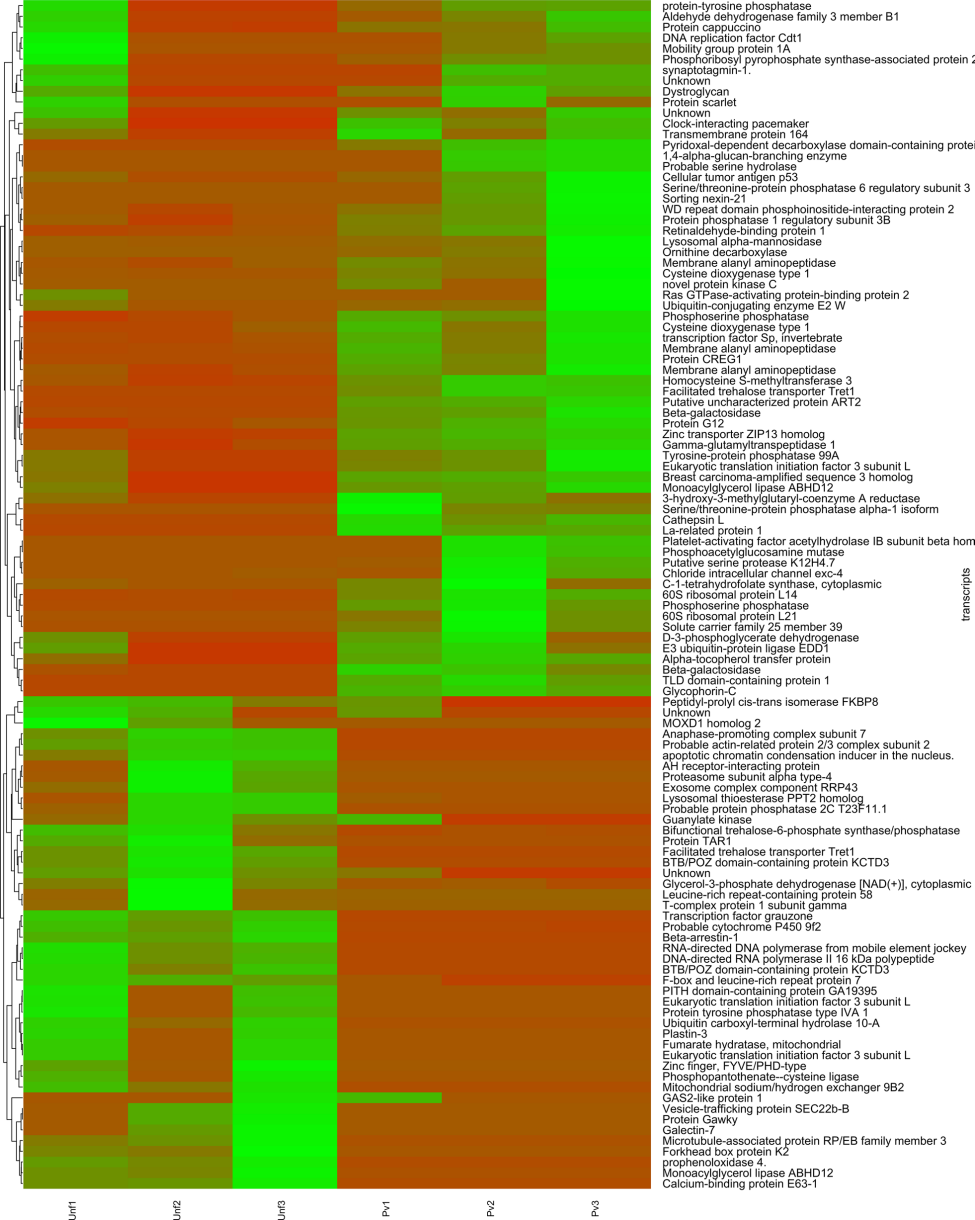
Heatmap showing differential expression of genes between *Plasmodium vivax-*infected (*Pv*) *versus* unfed mosquito (*Unf*) groups. Heatmaps were performed with normalized expression values using the *gplots* v.3.0.1 package in R

To examine differential expression between mosquitoes fed on *P. vivax* infected-blood (*Pv*) and mosquitoes fed on non-infective blood (*Bl*) or unfed mosquitoes, FDR < 0.05 and logFC > 1 (fold change) were used as the threshold to classify differentially expressed genes. The analyses showed a total of 12,942 expressed genes. Of these, 49 genes were identified as differentially expressed genes in the *P. vivax* infected-blood-fed group (*Pv*) in relation to non-infected-blood-fed group (*Bl*); 34 were upregulated and 15 were downregulated, which represents differential expression associated to *P. vivax* infection (Fig. 1). A total of 111 genes were differentially expressed in infected-blood-fed mosquitoes (*Pv*) when compared to the unfed group (*Unf*); of these, 65 were upregulated and 46 were downregulated (Fig. 2). The detailed gene lists are shown in Additional file 2: Tables S2–S5.

**Fig. 3:**
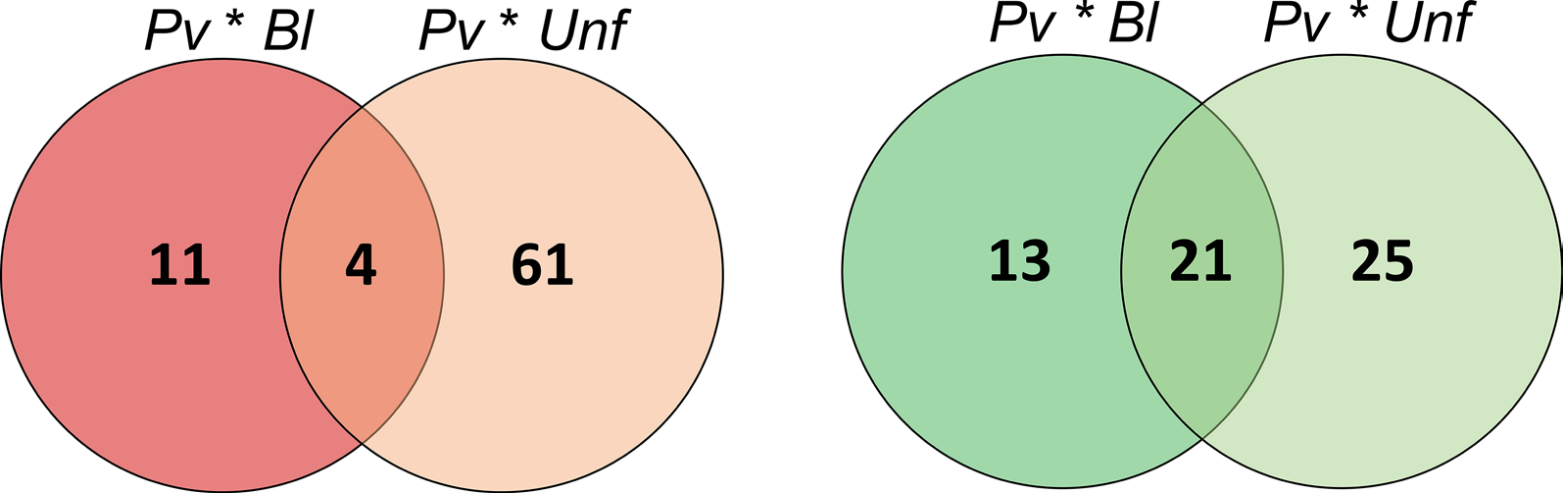
Proportions of *Anopheles aquasalis* midgut differentially expressed genes within different groups comparison. **a** Upregulated genes. **b** Downregulated genes. *Abbreviations*: *Pv*, *P. vivax* blood-meal group; *Bl*, non-infective blood-meal group; *Unf*, unfed group

About 49% (24 out of 49) of the differentially expressed genes in the *Pv* × *Bl* group were exclusive to this comparison (Fig. 3) and involved a large gene set related to autophagy. On the other hand, the *Pv* × *Unf* comparison presented 86 out of 111 (78%) of the differentially expressed genes exclusively in this group (Fig. 3).

To validate the robustness of RNAseq results, we analysed eight genes by real-time qRT-PCR and compared the expression of these genes in *Pv* × *Bl* (Additional file 3: Figure S1). These analyses revealed a significant correlation (Pearsonʼs correlation coefficient = 0.874*, R*^2^ = 0.7663, slope = 0.04539) between the qRT-PCR and the RNAseq data.

### *Anopheles aquasalis* midgut differential gene expression associated with *Plasmodium vivax* infection

**Fig. 4:**
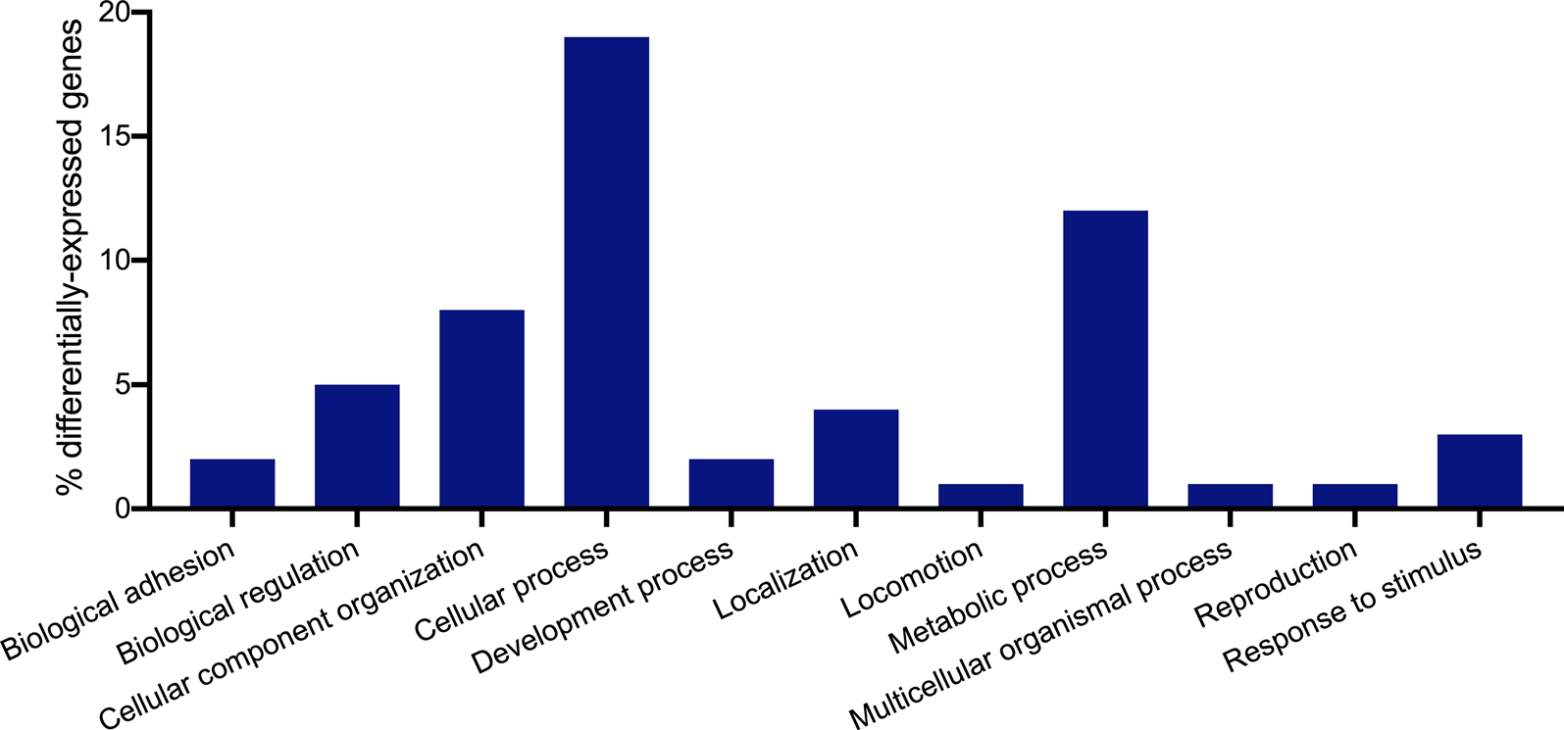
Functional classification of *Anopheles aquasalis* midgut transcripts using gene ontology analysis. Summary of the general distribution of differentially expressed genes [%] on PANTHER (http://www.pantherdb.org)

Transcriptomic analysis of midgut infected mosquitoes revealed 49 differentially expressed genes in the *P. vivax* infected-blood-fed group (*Pv*) in relation to non-infected-blood-fed group (*Bl*); of these, 34 were upregulated and 15 were downregulated. From these, genes involved in cellular process, metabolic process (GO: 0008152), cellular component organization or biogenesis process (GO: 0050896) and biological regulation process (GO: 0065007) were predominant (Fig. 4). The results suggest that many of the upregulated genes are involved in several metabolic processes and molecular functions, among them, catalytic activity (GO: 0003824) and cofactor binding (GO: 0005488) were enriched with hits of 6 and 4 genes, respectively, followed by transporter and structural molecule activity.

#### Immunity

Activation of mosquito immunity genes has been traditionally associated with midgut-infected mosquitoes. In the present study, a transcript coding for a leucine-rich repeat protein, orthologue of LRR-containing protein 58 (TRINITY_DN6165_c5_g1_i4), was found upregulated in mosquitoes infected with *P. vivax* (*Pv*) when compared to the mosquitoes fed on inactivated gametocytes (*Bl*). LRR-containing protein 58 has been previously associated with the *An. gambiae* response to *Plasmodium berghei* infection [38, 39]. LRR containing proteins are related to the control of the complement-like protein TEP1 function, and have other important roles in innate immune defence [40]. Information on all transcripts (TRINITY_DN0000_c00_g00_i00) are summarised in Additional file 2: Tables S2–S5.

#### Detoxification

*Plasmodium vivax* is probably able to modulate detoxification of free radicals while invading the midgut of *An. aquasalis*, as suggested by the increase of H_2_O_2_ following artificial reduction of catalase activity which leads to increased parasite infection in the mosquito midgut. As gene silencing also decreases the midgut microbiome, Bahia et al. [41] suggest that this manipulation occurs through the control of competitive bacteria which allows better parasite development. Transcript coding for CYP315A1 (TRINITY_DN6118_c3_g2_i14) was upregulated in the *Pv* × *Bl* group, as was its orthologue in deltamethrin-resistant *An. gambiae* mosquitoes when compared with a sensitive mosquito line from Kenya [42]. NAD+ kinase (TRINITY_DN5956_c5_g5_i5) and senecionine N-oxygenase (TRINITY_DN5975_c0_g1_i2) were also upregulated with infection, which suggests that the enzyme activity was needed for the antioxidant activities of other enzymes. Autophagic responses can be modulated by radical species and NAD+ homeostasis and the midgut metabolism can be an important player in autophagy regulation [43].

#### Cytoskeleton remodelling

*Anopheles gambiae* midgut response to *P. berghei* ookinete invasion is characterized by profound alterations in the transcription of genes that modulate the architecture of the cytoskeleton [38]. *Plasmodium* parasites need to modify the cytoskeleton of mosquito epithelial cells to successfully complete their life-cycle. We found several downregulated genes (TRINITY_DN4493_c0_g1, TRINITY_DN5277_c0_g1, TRINITY_DN5389_c0_g1, TRINITY_DN6055_c0_g1, TRINITY_DN6090_c5_g3, TRINITY_DN6296_c2_g1) that could be associated with cytoskeleton remodelling, which reinforces the prominent role of this cellular mechanism in response to *Plasmodium* and extends it to *P. vivax* ookinete invasion of the mosquito midgut.

#### Autophagy

The present transcriptional analysis suggested that differential expression of autophagic genes is involved in *An. aquasalis* females following a *P. vivax* infected blood meal. Forty-nine percent of differentially expressed genes during invasion (60.0% of the upregulated and 44.1% of the downregulated genes) could be associated with autophagic processes (Table 2).

**Table 2:** Midgut differentially expressed genes associated with autophagy upon *Plasmodium vivax* invasion

Transcript ID	Annotation	logFC
TRINITY_DN1272_c0_g1_i2	Protein spitz	8.52
TRINITY_DN4493_c0_g1_i2	T-complex protein 1 subunit gamma	− 9.19
TRINITY_DN5389_c0_g1_i2	gamma-tubulin complex component 5	− 8.22
TRINITY_DN5823_c0_g1_i1	Facilitated trehalose transporter-Tret1	− 9.50
TRINITY_DN5851_c0_g1_i2	GPCR family 2, methuselah receptor 6	− 3.20
TRINITY_DN5911_c0_g1_i4	Beta-arrestin-1	− 9.97
TRINITY_DN5956_c5_g5_i5	NAD kinase	9.26
TRINITY_DN6021_c0_g3_i1	OTU domain-containing protein 5	− 9.38
TRINITY_DN6039_c0_g1_i17	Ubiquitin-like-conjugating enzyme ATG3	− 9.27
TRINITY_DN6055_c0_g1_i13	Formin-like protein CG32138	− 11.08
TRINITY_DN6077_c7_g2_i1	Guanylate cyclase 32E	− 8.56
TRINITY_DN6090_c5_g3_i2	Probable actin-related prot. 2/3 complex subunit2	− 8.39
TRINITY_DN6177_c2_g2_i3	Trehalose-6-phosphate synthase/phosphatase	8.01
TRINITY_DN6296_c2_g1_i5	Plastin-3	− 9.74
TRINITY_DN6321_c0_g2_i2	Endothelin-converting enzyme 1	8.43
TRINITY_DN6330_c2_g6_i2	Fat storage-inducing transmembrane protein	− 8.49
TRINITY_DN6333_c5_g2_i4	Integrin beta-PS	8.32
TRINITY_DN6454_c2_g2_i6	Transcription factor kayak/AP-1	10.47
TRINITY_DN6473_c3_g4_i6	Dynein light chain 1, cytoplasmic	10.67
TRINITY_DN6489_c3_g1_i5	Prominin	3.40
TRINITY_DN6531_c1_g1_i4	Sphingomyelin phosphodiesterase	9.45
TRINITY_DN6536_c2_g8_i1	Ubiquitin carboxyl-terminal hydrolase 10-A	− 8.93
TRINITY_DN6571_c0_g10_i7	Forkhead box protein K2	− 10.44
TRINITY_DN6646_c8_g1_i10	Vesicle-trafficking protein SEC22b-B	− 6.81

Transcript coding for the GPCR Methuselah receptor 6 (TRINITY_DN5851_c0_g1_i2) was downregulated, as was β-arrestin (TRINITY_DN5911_c0_g1), which uncouples GPCRs from their G-proteins, and suggests that regulation of free radical production might occur through this molecule. In *Drosophila*, Methuselah receptors have been associated with lifespan and resistance to starvation and free radicals [44]. Wang et al. [45], using a specific agonist and antagonist, demonstrated that the TOR pathway is one of the major effectors underlying Methuselah. Blocking Methuselah reduced dTOR activity and promoted autophagy.

Trehalose is a natural sugar found in prokaryotes, yeast, fungi, plants and invertebrates, and serves not only as a reserve of carbohydrate, but can also protect organisms and cells against adverse environmental conditions. Some controversy exists on the real effect of trehalose on autophagy. In murine models, trehalose seems to induce autophagy, while in cultured cells it could inhibit fusions of autophagosomes and lysosomes, thus blocking the final stage of autophagy [46]. Our data suggest that the *An. aquasalis* midgut increases intracellular trehalose by upregulating trehalose 6-phosphate synthase/phosphatase (TRINITY_DN6177_c2_g2) and downregulating the TreT1-facilitated trehalose transporter (TRINITY_DN5823_c0_g1), suggesting autophagy induction in the *An. aquasalis* midgut during *P. vivax* infection. *Anopheles gambiae* TreT1 RNA silencing reduces the number of *P. falciparum* oocysts in the mosquito midgut [47], suggesting it might exert parasite protection during midgut invasion.

Microtubules (MT) are important to autophagosome formation and motility. Dynein light chain 1 (TRINITY_DN6473_c3_g4), a motor protein, was upregulated upon infection in our study. In vertebrates, Beclin-1 is sequestered in MT in complexes containing dynein light chain 1. When autophagy is stimulated, Beclin-1 is released from this complex. In parallel, c-Jun N-terminal kinase-1 (JNK1) is activated which allows phosphorylation of Bcl-2 and Bim, which, in turn, releases Beclin 1 and contributes to autophagosome formation [48]. Regarding the gamma-tubulin complex component 5 (TRINITY_DN5389_c0_g1), involved in microtubule assembly [49], and T-complex protein 1 subunit gamma (TRINITY_DN4493_c0_g1), a chaperonin for tubulin and actin [50], we found that these were downregulated, possibly confirming that (as in other Anopheline species) microtubule dynamics are altered during *P. vivax* invasion of the midgut epithelium, and it is possible that this is associated to autophagy.

JNK signalling has been demonstrated to be involved in lifespan control and is required in differentiated cells of the intestinal epithelium in order to prevent excessive sensitivity of these cells to oxidative stress in *Drosophila* [51] and has been implicated in mosquito defense against malaria parasites, and altered expression patterns of autophagy biomarkers [22]. Garver et al. [52] showed that basal mRNA expression of the genes involved in JNK signalling were upregulated in the mosquito midgut and JNK silencing significantly increases the prevalence of infection. However, more recently, Souvannaseng et al. [53] demonstrated that moderate inhibition of JNK signaling in the *An. stephensi* midgut extended lifespan and enhanced resistance to *P. falciparum*. In the present study, we found that the AP-1 transcription factor (TRINITY_DN6454_c2_g2), a downstream product of this signalling pathway, was upregulated in mosquitoes fed on *P. vivax* blood.

The ubiquitin machinery regulates fundamental biological processes within eukaryotic cells. The enrichment of functional terms such as ubiquitin-dependent proteasome was also denoted for insects facing dehydration stress [54]. Nitric oxide synthase expression and nitric oxide increase in the midgut of *An. aquasalis*, *An. stephensi* and *An. gambiae* during *Plasmodium* parasite infection, which limits parasite development within the mosquito [55–57]. Ubiquitin carboxyl-terminal hydrolase (TRINITY_DN6536_c2_g8), ubiquitin-like-conjugating enzyme ATG3 (TRINITY_DN6039_c0_g1) and OTU domain-containing protein 5 (TRINITY_DN6021_c0_g3) were downregulated during midgut invasion by *P. vivax*. OTU domain-containing proteins are deubiquitinating enzymes and cleave distinct sets of ubiquitin chain types [58]. In vertebrates, OTU domain-containing protein 5 regulates interferon signalling [59]. USP10 regulates the deubiquitination of Beclin1 in Vps34 complexes, which in turn leads to a reduction in the levels of PtdIns3P and consequent inhibition of autophagy. Spautin-1, an inhibitor of USP10 and USP13, promotes the ubiquitination and degradation of Vps34 complexes, which in turn leads to a reduction in the levels of PtdIns3P and consequent inhibition of autophagy [60]. Since ubiquitination and deubiquitination are central to autophagy regulation, once more, our data indicated that *P. vivax* invasion of *An. aquasalis* midgut epithelium triggers an autophagic response. ATG3, among other enzymes, is involved in the maturation of the growing autophagosome, a process that occurs once autophagy is initiated. Recently, Frudd et al. [61] described a mechanism that associates reactive species to autophagy induction. The oxidation of Atg3 and Atg7 prevents phosphatidylethanolamine conjugation to LC3 (microtubule-associated protein 1A/1B-light chain 3), thus, associating reactive species to autophagy induction. The production of reactive oxygen/nitrogen species during ookinete invasion of *An. aquasalis* [41], together with the set of differentially displayed genes such as, is suggestive of an interplay between reactive species and autophagy during ookinete invasion of the midgut.

Overexpression of prominin 1 constitutively activates autophagy in the human retinal pigment epithelium *via* inhibition of mTORC1 and mTORC2, while it impairs autophagy *via* upregulation of mTORC1/2 activities. Prominin (TRINITY_DN6489_c3_g1) was upregulated during parasite invasion of the midgut epithelium, which suggests that autophagy might be activated during this stage of infection.

Lipid droplet (LD) homeostasis [62] also plays an important role in autophagy regulation. We observed that a fat storage-inducing transmembrane protein (TRINITY_DN6330_c2_g6), that was described to facilitate proper LD budding from the ER [63], was downregulated. Sphingolipids have also been associated with lipid droplet formation, and sphingomyelin phosphodiesterase 1 (TRINITY_DN6531_c1_g1) was upregulated. Although sphingomyelinases do not play a role in autophagy induction, the upregulation of sphingomyelin phosphodiesterase 1 (TRINITY_DN6531_c1_g1) can act in the autophago-lysosomal degradation [64], thus regulating the autophagosome formation. Vesicle transport protein SEC22 (TRINITY_DN6646_c8_g1), a protein implicated in autophagosome biogenesis [65], was downregulated, while endothelin-converting enzyme (TRINITY_DN6321_c0_g2), previously detected in autophagic vesicles [66], was upregulated. Regulation of these genes suggests that *P. vivax* invasion affects regulation of different stages of the autophagic process and includes autophagosome maturation and degradation.

*Plasmodium* invasion of the midgut epithelial cell leads to a number of molecular and morphological changes, including cell death. Vlachou et al. [67] proposed that the first invaded cells undergo apoptosis and are expelled to the lumen, while adjacent cells extend lamellipodia to maintain epithelium continuity. The ookinete reinvades several cells until it reaches the extracellular matrix in order to develop into oocysts, which implies a substantial dynamic rearrangement of the actin cytoskeleton. Division of regenerative cells within the midgut epithelium of an adult female *An. stephensi* in response to *P. falciparum* invasion [68] has been reported. Even so, the extent of apoptosis and the mechanisms by which the integrity of the midgut epithelium is maintained are not yet understood. The detachment-induced apoptosis (anoikis), driven by these morphological changes, can be deleterious to the mosquito and can be compensated by extracellular matrix (ECM) detachment, which induces autophagy [69] mediated by integrin [70]. Integrin are cell surface proteins that interact with the external cellular matrix (ECM), and signal through the cell membrane in both directions. *Plasmodium vivax* infection of the midgut positively regulated the expression of this gene (TRINITY_DN6333_c5_g2).

The *Drosophila* epidermal growth factor receptor (EGFR) pathway has been implicated in the control of delamination and anoikis of damaged enterocytes following oral bacterial infection [71] and *Serratia marcescens* infection of *An. gambiae* activates the EGFR pathway by modulating the outcome, possibly through synergistic functions in gut homeostasis [72]. Spitz (TRINITY_DN1272_c0_g1), the ligand of EGFR, was upregulated with infection and Forkhead box K2 (TRINITY_DN6571_c0_g10_i7) which can inhibit EGRF in 769-P cells [73], which suggests that the EGFR pathway is activated following *P. vivax* infection. This probably contributes to gut hemostasis through autophagy. Subcellular localization of the EGFR seems to be determinant on the effect on autophagy, being either an inhibitor or stimulant [74].

### Treatment of mosquitoes with autophagy inhibitor

In order to evaluate the autophagy effect on the outcome of *P. vivax* infection in *An. aquasalis*, the mosquitoes were treated prior to infection with the autophagy inducer spermidine, or an autophagy inhibitor wortmannin.

**Fig. 5:**
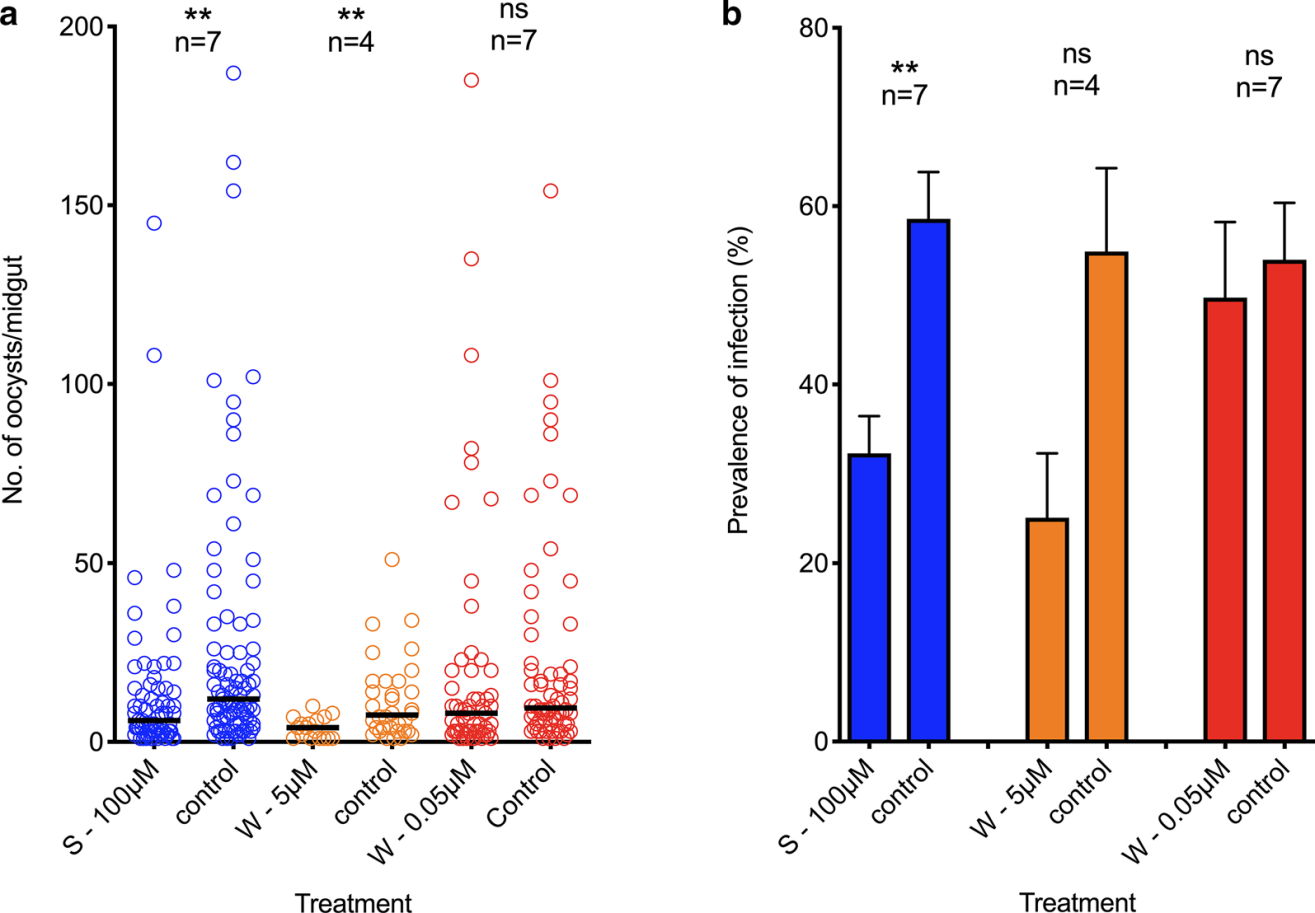
The effect of spermidine and wortmannin treatment during *Plasmodium vivax* infection of *Anopheles aquasalis*. **a** Infection Intensity. Dotted values represent individual oocyst counts/midgut. Horizontal lines represent the median number of oocysts per midgut. The Mann–Whitney U-test was used to compare the intensity of infection. **b** Prevalence of infection. t-test was used to compare average prevalence of infection. **P < 0.001. *Abbreviations*: ns, not significant; S, spermidine; W, wortmannin; n, number of independent experiments

When mosquitoes were treated with the autophagy inducer spermidine, the infection prevalence (IP) and infection intensity (II) were significantly lower: IP: Mann–Whitney U-test: *U* = 10196, *P* < 0.0001; II: Unpaired t-test: *t*_(12)_ = 3.913, *P* = 0.0021). A reduction of 44.9% (58.6 to 32.3%) in IP and of 47% in II (25.7 to 13.6%) was observed. Wortmanin treatment resulted in a 54.3% reduction in IP and a 65% reduction in II when higher doses were used, while the 0.05 µM doses resulted in a low reduction (7.9%) of IP and a 5.9% increase in II (IP: Mann-Whitney U-test: W-5 µM*control, *U* = 1351, *P* = 0.0002; W-0.05 µM*control, *U* = 7000, *P* = 0.2357); II: t-test: W-5 µM*control, *t*_(4)_ = 2,528, *P* = 0.0648; W-0.05 µM*control, *t*_(12)_ = 0.4003, *P* = 0.6960) (Fig. 5).

The differences between the two doses of wortmannin are probably a consequence of the drug mode of action. Wortmannin is a PI3-kinase inhibitor, therefore, as autophagosome formation requires class III PI3-kinase activity, it is normally used to study the effect of autophagy inhibition. Nonetheless, wortmannin can also inhibit class I PI3-kinase activity (which inhibits autophagy) and can also inhibit mTOR (an autophagy-inhibitory molecule) [75]. Furthermore, wortmannin can also act on the parasite and interfere with its development [76].

Spermidine is a polyamine that stimulates autophagy, both through mTOR-independent or dependent mechanisms [77] and its administration to mosquitoes produces a significant reduction in *P. vivax* infection after treatment. Polyamine biosynthesis inhibitors cause growth arrest of *P. falciparum* blood stages *in vitro* but show no effect on survival of mice infected with *P. berghei* (reviewed in [78]). Despite these data, polyamide biosynthesis seems to be fundamental for sporogonic cycle completion. Targeted deletion of the enzyme AdoMetDC/ODC from *Plasmodium yoelii* blocks transmission to the mosquito *An. stephensi*, which could not be rescued by supplementation with spermidine [79]. This information reinforces that spermidine is acting on the mosquito rather than the parasite. Our data demonstrated a significant reduction in *P. vivax* infection after spermidine treatment in *An. aquasalis*, which, together with the data obtained using low wortmannin treatment, suggests that autophagy can control *P. vivax* infection in *An. aquasalis.*

### Expression of autophagy genes following *Plasmodium vivax* infection and treatment with autophagy suppressor and inhibitor

To further characterize the role of autophagy in the mosquitoes treated with autophagy inhibitor in response to *Plasmodium* infection, qRT-PCR was used to quantify the changes in gene expression in response to a *P. vivax*-infected blood meal. A differential expression analysis of several autophagy genes, including *DRAM*, *Apg8* and *Beclin*, during inhibition and activation of autophagy, was performed. Atg8 protein, formerly known as Apg8/Aut7 is part of a group of proteins that control autophagy, many of which also participate in direct cytoplasm-to-vacuole transport of proteins [80, 81]. Among the genes that promote autophagy is the damage-regulated autophagy modulator (*DRAM*-1), which belongs to an evolutionarily conserved family of proteins that encodes for a lysosomal protein that is required in order to induce autophagy [82, 83], and Beclin-1, which is part of a Class III phosphatidylinositol 3-kinase complex that is thought to be important in mediating localization of other Apg proteins to pre-autophagosomal structures [60].

**Fig. 6:**
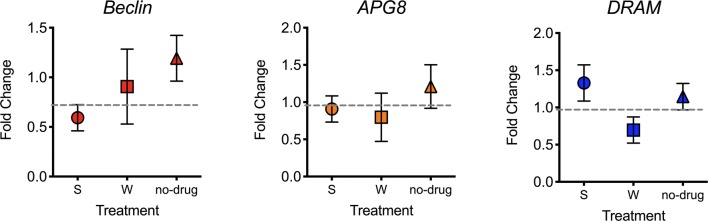
Expression of *Beclin*, *DRAM* and *Apg8* in response to *Plasmodium vivax* infection and treatment. *An. aquasalis* mosquitoes were treated with the autophagy inducer spermidine (S) and wortmannin (W) an inhibitor prior to infection and fed with *P. vivax* blood. Midguts were dissected (18–24 h) and *Beclin*, *Apg8* and *DRAM* abundances were measured by qRT-PCR and normalized to S7

No major differences were observed in expression of these genes after treatment with both drugs when compared with infection without treatment (Fig. 6). This is in-line with RNAseq data where transcription alterations of these genes were not detected. The major difference in expression was observed for *beclin*, which was downregulated after mosquitoes were treated with spermidine (*P* = 0.0635), suggesting that spermidine is downregulating this gene while exerting a negative effect on *P. vivax* sporogonic development.

## Conclusions

Our results clearly indicate that autophagy is regulated by *P. vivax* invasion of the mosquito midgut epithelium. A vast number of genes associated with autophagy were regulated by infection of which 60% were upregulated. Furthermore, when autophagy was inhibited by spermidine, we observed a significant reduction of the prevalence and intensity of infection. In view of our results, we propose that when ookinetes invade the midgut cells they trigger host cell morphological rearrangement, with actin and microtubule remodelling and production of nitrogen and oxygen radicals and possible cell death. To counterbalance invaded epithelial cell death/extrusion and other injuries parasites, could trigger an autophagic mechanism that would restrain parasite development, possibly through GPCR signalling Methuselah, the increase of intracellular trehalose, and detachment from the excellular matrix. This effect was apparent by the regulation of genes that could be assigned to the different stages of autophagy (initiation, nucleation, elongation/closure and maturation degradation) [84]. Autophagy triggered by *Plasmodium* invasion in epithelial midgut cells is a novel mechanism for mosquitos in order to fight *Plasmodium* infection.

## Additional files

**Additional file 1: Table S1.** List of primers used in qRT-PCR analysis.

**Additional file 2: Table S2.** Lists of differentially transcribed genes Pv × Bl_FDR < 0.05. **Table S3.** Lists of differentially transcribed genes Pv × Unf_FDR < 0.05. **Table S4.** Lists of differentially transcribed genes Pv × Bl_Total. **Table S5.** Lists of differentially transcribed genes Pv × Unf_Total.

**Additional file 3: Figure S1.** Validation of RNAseq analysis using qRT-PCR. Gene expression values for eight genes obtained by RNAseq were plotted against the corresponding averages of three qRT-PCR-derived gene expression values from biological replicates. The Pearsonʼs correlation coefficient (0.874) and the best-fit linear-regression analysis *R*^2^ = 0.7663 demonstrated a good degree of correlation between gene expression determined by each assay. Genes used for validation: TRINITY_DN4493_c0_g1_i2, TRINITY_DN5277_c0_g1_i2, TRINITY_DN5911_c0_g1_i4, TRINITY_DN6055_c0_g1_i13, TRINITY_DN6039_c0_g1_i17, TRINITY_DN6296_c2_g1_i5, TRINITY_DN6531_c1_g1_i4, TRINITY_DN6536_c2_g8_i1.

## Data Availability

The datasets supporting the conclusions of this article are included within the article and its additional files.

## Abbreviations

RNAseq: RNA sequencing
cDNA: complementary DNA
WHO: World Health Organization
ATG: translation of autophagy
MFA: membrane feeding assay
DMSO: dimethyl sulfoxide
PBS: phosphate buffered saline
RT-qPCR: quantitative reverse transcription PCR
