# Supplementary material for: Anopheles aquasalis transcriptome reveals autophagic responses to Plasmodium vivax midgut invasion
Source: Parasit Vectors. 2019 May 24;12:261. doi: 10.1186/s13071-019-3506-8 (PMC6534896; doi:10.1186/s13071-019-3506-8)
Supplement: Supplementary file 1 — Additional file 1: Table S1. List of primers used in qRT-PCR analysis. [file 13071_2019_3506_MOESM1_ESM.docx]

**Additional file 1: Table S1. List of primers used in qRT-PCR analysis**

| **Gene name** | **Assecion (Vectorbase)** | **Forward (5’-3’)** | **Reverse (5’-3’)** |
| --- | --- | --- | --- |
| S7 | GAMD01002944.1 | CCTGGAGGATTTGGTCTTCC | TGGTCTGCTGGTTCTTGTCC |
| **Validation of RNAseq analysis** | | | |
| TRINITY_DN4493_c0_g1_i2 |  | CCGAGGTGATCAAATCGTGT | GCATCGAGCGCTATCTTGAC |
| TRINITY_DN5277_c0_g1_i2 |  | TCTTCGTCCGTATTGGATGC | CTCATCTTGTCCCACCAGGA |
| TRINITY_DN5911_c0_g1_i4 |  | TGGAATCAGAGGATGGATGC | AGGCCCCACTTGTCTTTGTT |
| TRINITY_DN6055_c0_g1_i13 |  | ACAGCAGCAAAAGAGGACCA | TGTCGTTGGATTTGGTGTCA |
| TRINITY_DN6039_c0_g1_i17 |  | GATCCAAGCAACCTGGATGA | AGCCCACTCTCCTCGAACTC |
| TRINITY_DN6296_c2_g1_i5 |  | CCAGGGATCGTACAGTGGAA | GCTCGACCGCGTAGTTACAG |
| TRINITY_DN6531_c1_g1_i4 |  | GTCCGATTGTTTCAGCGTGT | GAACTGGGCACTGATGGTGT |
| TRINITY_DN6536_c2_g8_i1 |  | CTACCAACCAGCTGCTCCAG | ACATCCAACCGTTCTTCACG |
| **Autophagy related genes** | | | |
| BECLIN-1 | GAMD01002333.1 | ACCGATACCCTGCTCGAACT | ATCCGTGGTTTCCAGCTTCT |
| DRAM | GAMD01002749.1 | GCTGGTGAAGCAGAATCGTC | GCGACGAGCACTTTTTGTTC |
| APG8 | GAMD01002572.1 | CGGAAGACGCACTGTTTTTC | CTCCTCATGATGCTCGTGGT |
|  |  |  |  |
